# Surprising and novel multivariate sequential patterns using odds ratio for temporal evolution in healthcare

**DOI:** 10.1186/s12911-024-02566-4

**Published:** 2024-06-13

**Authors:** Isidoro J. Casanova, Manuel Campos, Jose M. Juarez, Antonio Gomariz, Bernardo Canovas-Segura, Marta Lorente-Ros, Jose A. Lorente

**Affiliations:** 1 AIKE research team (INTICO), Facultad de Informatica, University of Murcia, Campus de Espinardo, Murcia, 30100 Spain; 2 Murcian Bio-Health Institute (IMIB-Arrixaca), Murcia, Spain; 3 Department of Cardiology, Washington Hospital Center, Georgetown University, Washington, DC USA; 4 CIBER de Enfermedades Respiratorias, Instituto de Salud Carlos III, Madrid, Spain; 5 University Hospital of Getafe, Getafe, Spain; 6 European University of Madrid, Madrid, Spain

**Keywords:** Data mining, Knowledge discovery in databases, Odds ratio, Discriminative patterns, Sequential patterns, Interestingness measures, Burn units

## Abstract

**Background:**

Pattern mining techniques are helpful tools when extracting new knowledge in real practice, but the overwhelming number of patterns is still a limiting factor in the health-care domain. Current efforts concerning the definition of measures of interest for patterns are focused on reducing the number of patterns and quantifying their relevance (utility/usefulness). However, although the temporal dimension plays a key role in medical records, few efforts have been made to extract temporal knowledge about the patient’s evolution from multivariate sequential patterns.

**Methods:**

In this paper, we propose a method to extract a new type of patterns in the clinical domain called Jumping Diagnostic Odds Ratio Sequential Patterns (JDORSP). The aim of this method is to employ the odds ratio to identify a concise set of sequential patterns that represent a patient’s state with a statistically significant protection factor (i.e., a pattern associated with patients that survive) and those extensions whose evolution suddenly changes the patient’s clinical state, thus making the sequential patterns a statistically significant risk factor (i.e., a pattern associated with patients that do not survive), or vice versa.

**Results:**

The results of our experiments highlight that our method reduces the number of sequential patterns obtained with state-of-the-art pattern reduction methods by over 95%. Only by achieving this drastic reduction can medical experts carry out a comprehensive clinical evaluation of the patterns that might be considered medical knowledge regarding the temporal evolution of the patients. We have evaluated the surprisingness and relevance of the sequential patterns with clinicians, and the most interesting fact is the high surprisingness of the extensions of the patterns that become a protection factor, that is, the patients that recover after several days of being at high risk of dying.

**Conclusions:**

Our proposed method with which to extract JDORSP generates a set of interpretable multivariate sequential patterns with new knowledge regarding the temporal evolution of the patients. The number of patterns is greatly reduced when compared to those generated by other methods and measures of interest. An additional advantage of this method is that it does not require any parameters or thresholds, and that the reduced number of patterns allows a manual evaluation.

**Supplementary Information:**

The online version contains supplementary material available at 10.1186/s12911-024-02566-4.

## Introduction

In pattern mining, it is common to use the statistical significance of a pattern to reduce the huge number of patterns that are initially generated. A majority of these patterns are either unimportant or obvious, lacking the ability to provide novel insights to domain experts. To enhance the utility, relevance and usefulness of the patterns discovered, diverse measures of interestingness are employed to reduce their number [1].

A large number of specific quantitative indicators of test performance have been introduced into the clinical domain. These comprise specificity and sensitivity, likelihood ratios, area under the receiver operating characteristic curve (AUC), predictive values, and many more [2]. But there is a singular indicator named the Diagnostic Odds Ratio (DOR), which is an intuitive measure to clinicians for finding association between an exposure and an outcome and is closely intertwined with prevailing metrics. This indicator plays a pivotal role in enabling the formal meta-analysis of studies on diagnostic test performance and is derived through logistic regression [3].

In this paper, we propose a new data mining task [4] aimed at specifying the types of patterns or knowledge to be discovered during the data mining process in biomedical applications, where the diagnostic odds ratio can be calculated.

Therefore, we define Jumping Diagnostic Odds Ratio Sequential Patterns (JDORSP) and show how to use them in order to extract temporal knowledge regarding the evolution of the patients in the Intensive Care Burns Unit (ICBU).

We select these patterns by employing the DOR in a new way as regards its use as a measure to significantly reduce the number of patterns in the clinical domain and obtain only those sequential patterns with sound knowledge based on the definition of risk and protection factors.

The idea is to extract knowledge from a small number of sequential patterns that represent the patient’s state with a statistically significant protection factor, whose extensions (or evolutions) suddenly change the clinical state of the patient, thus making the patterns a statistically significant risk factor (or vice versa).

In addition to introducing JDORSP, we also evaluate the temporal knowledge they provide in the domain as regards two parameters: surprisingness and relevance for the domain. We additionally define a surprisingness measure with which to rank the patterns.

The remainder of this paper is organized as follows. Section 2 provides the literature review on the methods employed to discover discriminative patterns, the interestingness measures for data mining, including DOR, and the evolution of patients in an Intensive Care Burns Unit. Section 3 describes the methods used and the case study. Section 4 shows the experiments and provides a thorough discussion of the results obtained. Finally, we show our conclusions and future research.

## Literature review

### Discriminative patterns

In data mining, a pattern is considered significant when it meets certain criteria or thresholds that indicate its importance or usefulness. Discriminative pattern mining techniques have gained popularity due to their ability to uncover sets of significant patterns occurring with remarkable frequencies across class-labeled datasets. These methods facilitate the identification of meaningful patterns [5].

The study of discriminative patterns has advanced significantly, encompassing various non-uniform definitions such as contrast sets [6], emerging patterns [7] and subgroups [8, 9].

The process of discriminative pattern mining involves the assessment of pattern set frequency and utilizes statistical measures to evaluate the discriminative power of individual patterns or the complete pattern set [10]. Only those patterns or pattern sets that are able to pass the user-specified significance tests are considered significant.

Some researchers have carried out the mining process by adopting certain thresholds of the constraints, such as the growth rate [7, 11], the difference between the two supports [6, 12], information gain [13] or the odds ratio [14–16] to measure the discriminative power and then remove insignificant patterns.

Methods for the discriminative analysis of sequence data have also been proposed.

The mining of a minimal characteristic subsequence that occurs frequently in sequences of one class and infrequently in sequences of another has been studied by Ji et al. [17]. An efficient algorithm, denominated as ConSGapMiner, was designed in order to find all distinguishing subsequences. This algorithm follows a step-by-step process, encompassing candidate generation, frequency support computation, gap satisfaction testing, and post-processing techniques to ensure the removal of non-minimal outcomes.

In [18] the authors mined discriminative sequential patterns using significance threshold. First generate all the frequent sequential patterns using GSP, and the p-value of each frequent sequential pattern is calculated via the Fisher’s exact test. In addition, some patterns whose p-values are no less than the p-values of its sub-patterns are removed since these patterns are redundant.

Extracting concise and strong contrast information between two sequential datasets can be useful in the clinical evolution of patients, or in the construction of sequential classification models.

When discriminative patterns are used, one important question is how to select an appropriate measure in certain specific practical situations. Furthermore, Fang et al. [19] present an interesting formulation with which to divide discriminative patterns into several categories with respect to their different types of discriminative power. Notably, the efficacy of one discrimination measure may be different according to the targeted objectives, data types and discriminative pattern categories.

Choosing appropriate measures for discriminative power evaluation, therefore, sometimes requires domain knowledge and a clear acknowledgement of the nature of problems and data.

### Interestingness measures

The generation of rules from association rule mining or from discriminative pattern mining usually produces a huge set of rules that are impossible for domain specialists to manage. Moreover, these rules are generally superfluous because they vary slightly from each other, and their redundancy reduces the efficiency of the discovery. This is useless when the users have to sift through thousands or even millions of rules, because they lose the opportunity to interpret the results, find interesting rules or even use them to build a more accurate classifier [20].

Users are interested only in and are able only to evaluate from tens to a few hundreds of patterns. In order to solve this problem, interestingness measures should, therefore, be used to filter or to rank patterns and present a small number of patterns to users.

Diverse interestingness measures are widely employed across machine learning, data mining, and statistics domains. However, there is still no formal definition of “interestingness”. In their study, Geng and Hamilton [15] presented a comprehensive analysis of pattern interestingness, encompassing 9 essential criteria: conciseness, generality, reliability, peculiarity, diversity, novelty, surprisingness, utility and actionability.

These criteria may have overlaps or conflicts with others. For example, a concise pattern is, because of its simplicity, usually general, and generality may also lead to reliability. On the other hand, generality conflicts with peculiarity and novelty.

The default interestingness measures universally used in order to discover relevant association rules are support and confidence. The support-confidence framework is the most common framework used in most association rule mining methods and in order to mine and select rules for discriminative patterns [20].

Other studies use certain syntactical definitions to remove redundancies: for example, productivity [21], closure [22], constraints [23] or relevance [24].

Although support and confidence are, in many cases, appropriate measures with which to build a strong model, they are still not the ideal measures. The choice of a minimum support threshold in data mining requires careful consideration. A high threshold risks capturing only self-evident knowledge, missing out on exceptional cases that are interesting. Conversely, a low threshold yields a vast number of rules, often redundant or noisy, making it challenging to effectively calibrate the support setting [20].

There are several papers in which the authors compare different interestingness measures. For example, in [25] the authors investigate sixty-one objective interestingness measures such as support, confidence, conviction, lift, leverage, gini index or chi-square, among others to analyze their similarity and dissimilarity as well as their relationship.

In addition to these related works, we refer the reader to McGarry [26] and Geng and Hamilton [15] for more general information about interestingness measures.

### Evolution of patients in intensive care burns units

Intensive Care Burns Units (ICBU) are specialized healthcare facilities dedicated to the treatment of severe burn injuries, usually with inhalation injuries.

The initial evaluation and resuscitation of patients with extensive burn injuries, necessitating hospitalization, can be only loosely guided by formulas and rules [27]. However, the inherent limitations and inaccuracies of these formulas mandate continuous re-evaluation and adjustment of fluid infusions based on resuscitation goals. Key factors such as patient incomings, diuresis, fluid balance, acid-base balance (pH, bicarbonate, base excess), among others, are essential for defining objectives and monitoring the progression and response to treatment.

Nevertheless, the evaluation of these parameters is essential not only during the critical resuscitation phase (initial 2 days) but also throughout the subsequent stabilization phase (consecutive 3 days), as it contributes to a comprehensive understanding of patient progression and treatment effectiveness.

It might be possible to discover interesting multivariate sequential patterns that could help clinicians provide new insights concerning their patients’ evolution.

Furthermore, the ability to predict early mortality following admission is crucial in determining the appropriate course of action, whether it be an aggressive or conservative therapeutic approach. In a previous paper [28], we considered the patients’ evolution as regards mortality prediction by using emerging patterns with a knowledge-based temporal abstraction and then building highly sensitive and specific patient-survival classifiers.

## Methods

### Sequential patterns

Let I = {i_1_, i_2_, …, i_k_} represent a set of items. An itemset t is a non-empty subset of I. A sequence s = ⟨t_1_, t_2_, …, t_m_⟩ is an ordered list of itemsets (t_i_ ⊆ I) (also referred to as elements or events). The items within an element are unordered and are listed alphabetically. An item can occur at most once in an element of a sequence but may appear multiple times in different elements of a sequence. Multivariate sequences are sequences that have multiple attributes for each item in the sequence.

The length of a sequence is determined by the number of instances of items it contains. A sequence with a length of k is denoted as a k-sequence. For example, s = ⟨a, bce, de, bcde, f⟩ is a sequence comprising 6 distinct items {a, b, c, d, e, f} and 5 itemsets. The length of this sequence is 11.

Each itemset within a sequence represents the set of events occurring simultaneously (at the same timestamp). Different itemsets may appear at different times, but not necessarily the following day.

A sequence u = ⟨a_1_, a_2_, …, a_n_⟩ is considered a subsequence of sequence e = ⟨b_1_, b_2_, …, b_m_⟩ (or e is a supersequence of the sequence u), denoted as u ⪯ e, if integers i_1_ < i_2_ < … < i_n_ exist, such that a_1_ ⊆ b_i1_, a_2_ ⊆ b_i2_, …, a_n_ ⊆ b_in_. For instance, ⟨a, bce, f⟩ is a subsequence of s.

Given a sequence database D = ⟨s_1_, s_2_, …, s_n_⟩, the support of a sequence s ⊆ D is defined as the number of sequences in D that contain s. If the support of a sequence s satisfies a pre-specified minimun support threshold, s is considered a frequent sequential pattern.

We employed the FasPIP mining algorithm [29], which utilizes the Equivalence Classes Strategy and is able to discover multivariate sequential patterns represented by time points. This representation incorporates three distinct time operators (<, =, >), to establish relationships between points, indicating occurrences before (<), simultaneous or co-occurring (=), and after (>) each other. Furthermore, since the “after” operator (>) is the inverse of the “before” relation (<), considering a relation from the first occurring point obviates the need for the “after” operator. For example, a > b can be expressed as b < a.

During the candidate generation phase, FasPIP employs two distinct operations to expand a sequence by adding an item, thereby creating a new sequence: Sequence extensions, where frequent points occur after the existing sequence, and Itemset extensions, where points occur simultaneously with the last item in the pattern. For example, considering the sequence α = ⟨a < b⟩ and an item c ∈ I, the sequence β = ⟨a < b < c⟩ represents a Sequence extension (S-extension), while γ = ⟨a < b = c⟩ denotes an Itemset extension (I-extension).

### Jumping emerging patterns

Emerging Patterns (EP) [7] refer to sets of item conjunctions with attribute values that exhibit significant frequency changes across different datasets. Mining Emerging Patterns involves the task of identifying patterns (itemsets) whose growth rates (the ratio of their frequency between two classes) surpass a given threshold [1].

Moreover, a Jumping Emerging Pattern (JEP) [30] is an EP that exhibits an infinite growth rate, meaning it appears in one class but not in the other.

### Diagnostic odds ratio as interestingness measure in the clinical domain

In various clinical contexts, clinicians heavily depend on the accurate interpretation of diagnostic data (see Table 1). A wide range of tests have been proposed with the aim of improving diagnostic decision-making in diverse clinical scenarios.

**Table 1:** 2 × 2 Contingency table. The abbreviations TP, FP, FN, and TN respectively denote the number of true positives, false positives, false negatives, and true negatives

Diagnostic test	Disease Present	Disease Absent
Positive Negative	TP FN	FP TN

For example, accuracy can be expressed by sensitivity (proportion of positives among people with disease) (see Eq. 1) and specificity (proportion of negatives among people without disease) (see Eq. 2).

1 $$sensitivity=\frac{TP}{TP+FN}$$

2 $$specificity=\frac{TN}{TN+FP}$$

In Glas et al. [3], the adoption of the Diagnostic Odds Ratio (DOR) as a single indicator of diagnostic performance is suggested. The DOR serves as a measure of the discriminative capability of a diagnostic test, representing the ratio of the odds of a positive test result among the diseases to the odds of a positive test result among those without the disease (refer to Eq. 3).

3 $$DOR=\frac{\frac{TP}{FN}}{\frac{FP}{TN}}=\frac{\frac{sensitivity}{1-sensitivity}}{\frac{1-specificity}{specificity}}$$

The DOR is not prevalence dependent, and may be easier to understand, as it is a familiar epidemiological measure and has, therefore, been widely used in health and medical practice and research.

The DOR takes values ranging from 0 to infinity. The further the DOR is from 1, the more likely it is that those with the disease are exposed when compared to those without the disease (risk factor). A DOR of 1 indicates that the test does not differentiate between patients with and without the disease. Values below 1 suggest a decreased risk of disease association with exposure (protection factor).

Conventional calculation of confidence interval (CI) for range estimates is commonly performed as depicted in Eq. 4, where Xhm represents the Mantel-Haenszel chi-square and Z = 1.96 is utilized for a 95% confidence level. In practice, the 95% CI is frequently employed as an indicator of statistical significance if it does not overlap with the null value (OR = 1).

4 $$\eqalign{& CI = DO{R^{(1 \pm {Z \over {Xhm}})}},\cr Xhm  & = \sqrt {{{(n - 1){{(TP \times TN - FP \times FN)}^2}} \over {(TP + FP)(FN + TN)(TP + FN)(FP + TN)}}} \cr}$$

Another statistical metric often used in epidemiological studies is relative risk. The DOR and relative risk are consistent. A larger diagnostic odds ratio leads to a larger relative risk, and vice versa. Under the rare-disease assumption, the odds ratio approximates the relative risk.

In [31], and later in [32], the authors use relative risk as a measure of the interestingness of patterns, defining risk patterns and excluding superfluous patterns.

In a recent article [33], the authors argue against the primary use of the relative risk ratio in clinical research. They assert that the relative risk’s direct interpretation lacks meaning and propose replacing it with the DOR. According to their findings, the DOR measures solely the effect magnitude and has no relationship to the prevalence of an outcome in a study nor does it overestimate the relative risk as is commonly thought.

Another statistical measure used in rule discovery is Chi-square [6], although, in general, any statistical test with a significant p-value could be employed [21, 34]. A number of these measures do not indicate the strength of the association. They are inappropriate for comparing values of quality of two subgroups and unsuitable for choosing top subgroups. In contrast, the DOR indicates the strength of an association [35].

It should also be noted that in [36] the authors illustrate that traditional statistical methods used by epidemiologists to assess etiologic associations are not adequate to determine the potential performance of a test for classifying or predicting risk for persons. This implies that the discriminatory power of the DOR is often questioned [37]. Since an odds ratio is a single number, it does not account for the trade-off between accurately identifying sick patients and mistakenly identifying otherwise healthy individuals. However, its association with the relative risk has long made it valuable for characterizing population variations in risk.

### Using the non overlapping of the confidence interval of the DOR

In Li et al. [14], a technique for eliminating redundant rules was introduced, utilizing the overlap of confidence intervals of the Diagnostic Odds Ratio (DOR). As shown in the previous section, the DOR is typically accompanied by its 95% confidence interval (CI) to demonstrate the accuracy of the estimate. The authors employed confidence intervals to determine whether a rule and its extension are statistically different. Non-overlapping confidence intervals indicate that the rules must carry different information, while overlapping intervals suggest the equivalence of the rules, leading to the pruning of the extension.

Several works based in the non-overlapping of DOR have subsequently been produced. In Toti et al. [38], the authors discuss the differences in performance obtained when extracting rules with the different definitions of non-exposed population, with no pruning criteria used to filter redundant rules, or when adding a pruning criterion of redundant rules based on an overlapping of 95% CI. They observed that mining without any pruning criteria resulted in a significant number of redundant rules, highlighting the necessity for an elimination process.

In another work [39], the authors emphasize the necessity of replacing traditional interest metrics such as support and confidence with metrics that specifically address the variations in risk resulting from different exposures. They propose two post-processing pruning criteria for refining the rule set. Firstly, a rule is pruned if its 95% CI for the DOR intersects with the value of 1. Secondly, a rule is pruned if its 95% CI does not overlap with any of its parent rules. The algorithm employed in their study successfully identifies interesting patterns among numerous combinations; however, the interpretation of the output still requires domain expertise.

### Case study

In this work, we analyze a clinical dataset comprising 480 patient records collected between 1992 and 2002 from the Intensive Care Burns Unit (ICBU) at the University Hospital of Getafe in Spain.

Firstly, we considered only those patients who survived during the period studied and those for whom it was possible to estimate the hours of hospital stay. After this cleansing, 465 patients remained, of which 81.29% (378/87) eventually survived, 69.68% (324/141), were male, and 43.23% (201/264) had inhalation injuries. Table 2 provides a summary of the static attributes of the database.

**Table 2:** Attribute summary

Attribute	Min	Max	Media	Std. Dev.
Age (years)	9	95	46.42	20.34
Weight (kg)	25	120	71.05	10.77
Length of stay (days)	3	162	25.02	24.24
Total burn surface area (%)	1	90	31.28	20.16
Deep burn surface area (%)	0	90	17.01	17.41
SAPS	6	58	20.67	9.49

We extracted six time series of both laboratory and physiologic data from the health records. The registered variables recorded during five days were: a) total of managed liquids measured in cc (INC); (b) diuresis in DC (DIUR); (c) balance of fluids in DC (BAL); (d) pH (PH); (e) bicarbonate in mmol/L (BIC); and (f) base excess in mEq/L (BE). All the attributes are continuous variables that represent cumulative values recorded over a 24-hour period. It is important to note that the fluid balance attribute (BAL) is not the difference between incomings (INC) and diuresis (DIUR); rather, it includes all potential eliminations of fluids, ensuring a comprehensive assessment of the fluid dynamics within the patient’s system.

### Three step knowledge discovery process

In our previous paper [28], models with which to predict mortality in ICBU were built by defining a 4-step knowledge discovery process. The initial two steps of our methodology concentrate on pre-processing the database and applying a pattern discovery technique to show the patients’ progression. Subsequently, we introduce a post-processing step to reduce the number of identified patterns. Lastly, to achieve interpretable models, we utilize the remaining patterns to construct classification models, which can take the form of rules or decision trees.

**Fig. 1:**
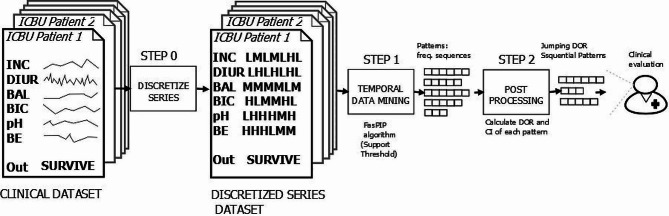
3-step knowledge discovery process for JDORSP

In this experiment, we have employed the first three steps, because we wish only to obtain a reduced number of sequential patterns with a specific medical behavior, and do not intend to use these patterns to build a classification model. Figure 1 illustrates this 3-step knowledge discovery process.

#### Step 0: “Discretization of temporal attributes”

In step 0, “Discretization of temporal attributes”, we used the discretization generated by an expert on the basis of clinical reference values, along with the Unsupervised Correlation Preserving Discretization (UCPD), for every attribute. These measures were selected according to a previous work [40].

On the one hand, the reference range discretization carried out by an expert (see Table 3) was determined from a variety of sources, and on the other, automatic cut points computed by employing the UCPD discretization method are shown in Table 4.

**Table 3:** Cut points of each attribute when using Expert discretization

Cut point	INC	DIUR	BAL	BIC	pH	BE
First	2.3	0.5	-2.0	17.0	7.20	-4.0
Second	3.66	1.0	10.5	21.0	7.30	-2.0
Third	5.78	1.9	20.4	25.0	7.35	2.0
Fourth			52.22	29.0	7.45	4.0
Fifth					7.50	
Sixth					7.60	

**Table 4:** Cut points of each attribute when using UCPD discretization

Cut point	INC	DIUR	BAL	BIC	pH	BE
First	0.2303	1.4941	0.5701	16.5	7.2767	-7.8833
Second	0.2913	1.7749	4.7804	17.6667	7.29	-6.63333
Third	0.3203	2.0857	7.4763	19.6167	7.3667	-4.3167
Fourth	0.5588	2.1697	13.1833	23.0333	7.3983	-0.2333

An expert discretization is preferred by clinicians because it is based mainly on reference range values, and it is necessary to interpret the patterns manually. For a better understanding of patterns, intervals of expert discretization are shown in Table 5. For example, if i marks the i discretization interval where i = 0 is the lowest interval, the item pH_0_ means severe acidosis [<, 7.20), pH_1_ = moderate acidosis [7.20, 7.30), pH_2_ = mild acidosis [7.30, 7.35), pH_3_ = normal [7.35, 7.45), pH_4_ = mild alkalosis [7.45, 7.50), pH_5_ = moderate alkalosis [7.50, 7.60], and pH_6_ = severe alkalosis [7.6, >).

**Table 5:** Discretization intervals of each attribute using expert discretization

Interval	INC	DIUR	BAL	BIC	pH	BE
0	[0, 2.30)	[0, 0.5)	[-, -2.0)	[-, 17)	[-, 7.20)	[-, -4)
1	[2.30, 3.66)	[0.5, 1.0)	[-2.0, 10.5)	[17, 21)	[7.20, 7.30)	[-4, -2)
2	[3.66, 5.78)	[1.0, 1.9)	[10.5, 20.4)	[21, 25)	[7.30, 7.35)	[-2, 2)
3	[5.78, -)	[1.9, -)	[20.4, 52.22)	[25, 29)	[7.35, 7.45)	[2, 4)
4			[52.22, -)	[29, -)	[7.45, 7.50)	[4, -)
5					[7.50, 7.60)	
6					[7.60, -)	

#### Step 1: “Mining multivariate sequential patterns”

In step 1, “Mining multivariate sequential patterns”, we use the FasPIP algorithm [29].

We have considered different rule supports depending on the discretization from 16% to 6% in order to generate the patterns, as in Casanova et al. [40], in which we compared different discretization algorithms in an attempt to discover the highest support that generates the lowest number of patterns, spans to the 5 days, and produces the best classification results. This will, therefore, allow us to compare the number of patterns obtained and observe the reduction attained.

**Fig. 2:**
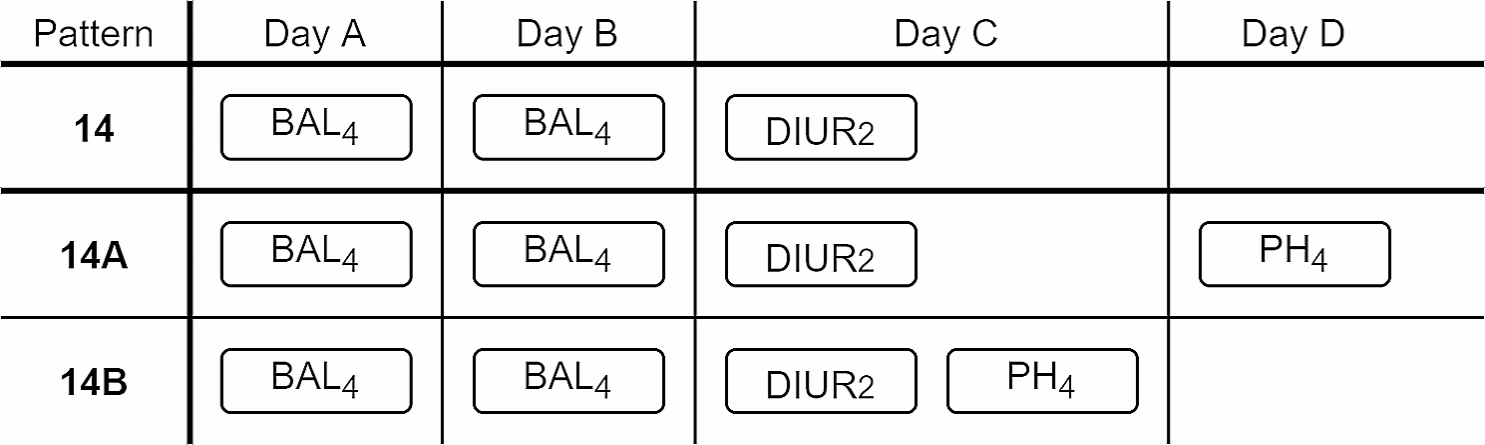
Example of pattern extensions

For example, the pattern number 14 (BAL_4_ < BAL_4_ < DIUR_2_) (172 patients) (extracted from Appendix A) with a length of 3 items on three different days A, B, and C, was found by using expert discretization. This temporal sequence starts with balance of fluids over 52.22 (BAL_4_) for two consecutive days (A and B), and it is followed (on day C) by diuresis between 1.0 and 1.9 (DIUR_2_). Figure 2 shows this pattern and its extensions with a new item (PH_4_). First the s-extension number 14A (BAL_4_ < BAL_4_ < DIUR_2_ < PH_4_) (45 patients), on another day D, where Day A < Day B < Day C < Day D, and second the i-extension number 14B (BAL_4_ < BAL_4_ < DIUR_2_ = PH_4_) (54 patients), on the same day C. Note that the days in the sequence are not necessarily consecutive.

#### Step 2: “Post-processing”

Finally, in step 2, “Post-processing”, we select the Jumping DOR Sequential Patterns on the basis of the DOR definition of the risk and protection factors. These new patterns are explained in more detail in the following section.

To compute the DOR, we address the potential issues arising from sensitivities and specificities of 100% by adding 0.5 to all cells in the diagnostic contingency table.

### Jumping DOR sequential patterns (JDORSP)

As we explained in Sect. 3.3, when we calculate the DOR of each pattern, we also can calculate the confidence interval in order to infer whether or not the association is statistically significant. Confidence intervals play a crucial role in the interpretation of the DOR in terms of both clinical significance and statistical significance.

We have chosen a 95% confidence interval (CI), which shows whether a DOR is statistically significant [41]:

- When the entire 95% CI is below 1, it indicates statistical significance in the DOR, suggesting a protective effect of the exposure in the study population.
- When the entire 95% CI is above 1, it indicates statistical significance in the DOR, suggesting a significant risk associated with the exposure in the study population.
- When the 95% CI overlaps DOR = 1, the DOR is said to be not statistically significant in the study population. This may reflect a true absence of a relationship between the exposure and the disease.

We propose a new way in which to use the DOR to reduce the number of patterns. We choose only the i-th pattern p_i_ with length (l) items at a specific point in time (t), that has a statistically significant *protection factor (DOR and CI < 1)*, and its n extensions (p_i1_, p_i2_, ..., p_in_) of p_i_ with length (l + 1) items, which could be an S-extension (in the next time, t + 1) or an I-extension (in the same time, t), that have a statistically significant *risk factor (DOR and CI > 1)*, and vice versa.

Besides, we can select “all” the pattern extensions or choose only the “best” pattern extension by using a beam search for the highest (or lowest) value of DOR.

**Fig. 3:**
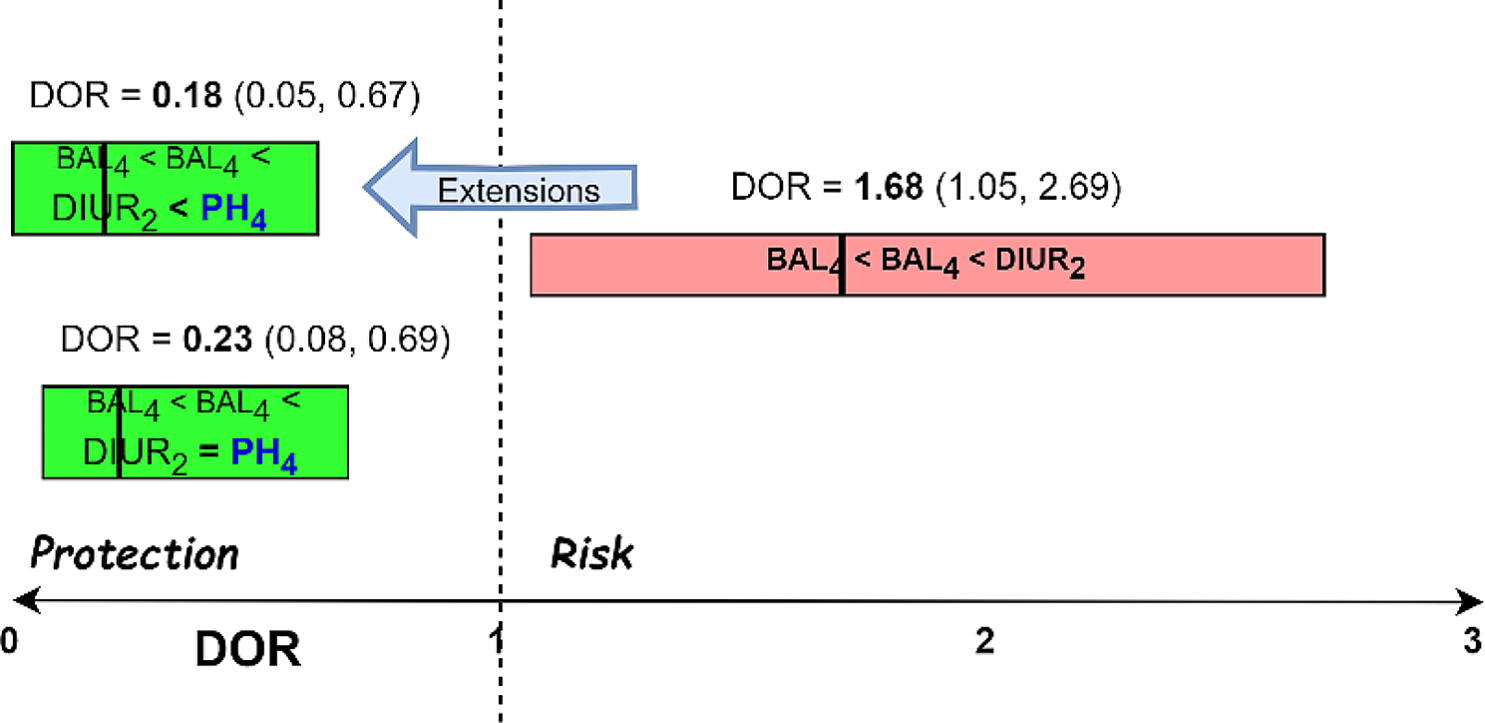
Example of jumping DOR sequential patterns

For example, Fig. 3 shows pattern number 14 (BAL_4_ < BAL_4_ < DIUR_2_) studied previously in Fig. 2 and extracted from Appendix A, with a length of 3 items and DOR value of 1.68 in the interval (1.05, 2.69), having a statistically significant risk factor (DOR and CI > 1), and then its 2 extensions, with a statistically significant protection factor (DOR and CI < 1) and a length of 4 items, first the S-extension number 14A (BAL_4_ < BAL_4_ < DIUR_2_ < PH_4_), with a DOR of 0.18 in the interval (0.05, 0.67) and second the I-extension number 14B (BAL_4_ < BAL_4_ < DIUR_2_ = PH_4_), with a DOR of 0.23 in the interval (0.08, 0.69). If we wanted to select the “best” protective extension, we would choose only pattern 14A, as it has the smallest DOR value, being more protective. Note that the entire 95% confidence interval of pattern 14 is greater than 1, and its extensions (14A and 14B) have an entire 95% confidence interval less than 1.

This makes it possible to obtain sequential patterns with the following interpretation or meaning: at a particular point in time, it is possible to state that the patient will survive (with a statistically significant DOR protection factor), however, something suddenly happens (usually the next day), and a pattern extension has a statistically significant DOR risk factor (or vice versa).

We denominate this as Jumping Diagnostic Odds Ratio Sequential Patterns (JDORSP).

### Proposed ranking measure: surprisingness score

In order to create a ranking with which to prioritize the patterns according to their interest, we define a measure that is based on the difference in DOR between a pattern and its extensions. The bigger the difference, the more surprising the pattern is.

After obtaining all the extensions of a pattern, we could rank them with this new measure. In our case, we wish to prioritize the most surprising patterns, so we define surprisingness score, SUR, as the maximum of the difference in the absolute value of a pattern with any of its extensions. Let p be a pattern, and let P_x_ be the set of all the extensions of pattern p. SUR is formally defined in Eq. 5.

5 $$\eqalign{{\rm{SUR = }} & {\rm{max(}}\left| {{\rm{dor}}(p) - dor(x)} \right|{\rm{)}} \cr & \left\{ {{\rm{x}} \in {{\rm{P}}_{\rm{x}}}} \right\} \cr}$$

## Experiments and discussion

We shall choose only those sequential patterns that have a DOR with a statistical protection factor and all the consecutive patterns with a statistical risk factor, and vice versa (these are denominated as Jumping DOR Sequential Patterns).

In order to compare the number of patterns generated, we propose two baseline experiments with which to select discriminative patterns that do not need user-specified thresholds either: using Jumping Emerging Patterns (JEP) and using the non overlapping of the DOR. There is a discussion of the results of these baseline experiments in our previous paper [16].

### Baseline experiment 1: using Jumping Emerging Patterns (JEP)

In our initial baseline experiment, we focused on identifying discriminative patterns, which are regarded as a fundamental technique in data mining [10]. To ensure a comprehensive analysis of both survivors and non-survivors, we performed pattern extraction separately on the subset of survivors and the subset of non-survivors. This approach allowed us to eliminate any common patterns representing typical patient evolution and focus solely on patterns that exhibited discriminatory characteristics between the two groups.

### Baseline experiment 2: using the non overlapping of the confidence interval of the DOR

In the second baseline experiment, we selected the patterns with a statistically significant change in the DOR, as stated in Li et al. [14]. The diagnostic odds ratio between two patterns is significantly different if their 95% confidence intervals do not overlap. In addition, only rules with an interval that does not cross 1 have been included in the output (as occurred in Toti et al. [39]). All the rules will, therefore, be statically significant.

### Experiment: using Jumping DOR Sequential Patterns (JDORSP)

Finally, we selected the new Jumping DOR Sequential Patterns that have been proposed in Sect. 3.7.

Note that by the definition of JDORSP, they are a subset of the patterns extracted in baseline experiment 2, i.e., JDORSP ⊆ non_overlapping_CI_DOR, since there will never be an overlap of a pattern with its extension, when transitioning from risk to protection (or vice versa).

The patterns obtained have the highest quality of all the experiments carried out previously because there are very few patterns, and they can, therefore, be manually reviewed by an expert in order to evaluate their possible clinical relevance.

### Results of the experiments

Table 6 depicts the number of discriminative patterns that have been selected after processing the two baseline experiments and our new proposal using different discretization algorithms (Expert and UCPD) and varying the rule support depending on the discretization.

**Table 6:** Number of patterns selected from the subset of survivors and nonsurvivors after processing the two baseline experiments and our new proposal (JDORSP) using Expert and UCPD discretization

Discre- tization	Rule Support	Survivors + Non-survivors Initial Patterns	Baseline 1 JEP	Baseline 2 Non Overlap DOR		Experiment JDORSP	
				all	best	all	best
Expert	10%	46,041 + 83,015	391	858	746	83	76
	**8%**	**88,084 + 241,866**	**4,931**	**2,195**	**1,856**	**163**	**146**
	6%	224,952 + 492,504	47,113	4,545	3,803	303	273
UCPD	16%	238,337 + 49,947	2,179	1,529	1,415	269	212
	14%	396,238 + 68,654	7,556	2,296	2,052	357	280
	12%	647,943 + 137,546	22,940	6,418	5,228	680	552

As explained previously, we have carried out two kinds of experiments in which the DOR between patterns was used to choose the discriminative patterns. We can select “all” the pattern extensions or choose only the “best” pattern extension by using a beam search for the highest value of DOR. This thus makes it possible to slightly reduce the number of patterns with baseline experiment 2 and with JDORSP experiment. In our case, few patterns have two or more extensions, but this criterion is generally valid.

As Table 6 shows, there is generally a great reduction in the number of patterns. This signifies that, for example, for expert discretization and 8% support, from 329,950 initial patterns (88,084 patterns for survivors + 241,866 patterns for non-survivors), we obtain 4,931 patterns using JEP (Jumping Emerging Patterns) (that is -98.5% of the original patterns that have been mined). With the non-overlapping of DOR (all), we obtain 2,195 patterns (-55.5% by using JEP), while when using our new Jumping DOR Sequential Patterns proposal (all), we obtain 163 patterns exclusively (-92.57% by using the non-overlapping of DOR).

Moreover, it will be noted that the reduction in the number of patterns is similar when using the UCPD discretization.

Furthermore, Table 7 shows the number of patterns initially selected and their number of extensions when the DOR is used to mine patterns. If we continue with expert discretization and 8% support (all), it is, therefore, possible to see that using the non-overlapping of the DOR makes it possible to obtain 2,195 patterns, of which 928 are initial patterns and 1,267 are their extensions. When an initial pattern has a DOR of protection, the extensions can move to risk (41 patterns) or continue with a protection factor (21 patterns). Moreover, the initial pattern can have a risk factor, and the extensions have a risk factor (1,156 patterns) or a protection factor (49 patterns).

**Table 7:** Number of patterns and all their extensions with the change of DOR factor for Expert and UCPD discretization (all)

Mining using DOR	Discretization	Rule Support	Number patterns			Number of extension patterns			
						init. *Prot*. to ext.:		init. *Risk* to ext.:	
			Initial	Extension	Total	*Risk*	*Prot*.	*Risk*	*Prot*.
Baseline 2 Non Overlapping DOR (all)	Expert	10%	373	485	858	14	21	419	31
		**8%**	**928**	**1,267**	**2,195**	**41**	**21**	**1,156**	**49**
		6%	1,901	2,644	4,545	86	21	2,456	81
	UCPD	16%	707	822	1,529	135	483	176	28
		14%	1,024	1,272	2,296	186	593	462	31
		12%	2,600	3,818	6,418	363	675	2,739	41
Experiment JDORSP (all)	Expert	10%	38	45	83	14	0	0	31
		**8%**	**73**	**90**	**163**	**41**	**0**	**0**	**49**
		6%	136	167	303	86	0	0	81
	UCPD	16%	106	163	269	135	0	0	28
		14%	140	217	357	186	0	0	31
		12%	276	404	680	363	0	0	41

As will be noted, with expert discretization and using the non-overlapping of the DOR, it is usual to start with initial patterns that are a risk factor to extensions that also constitute a risk factor. We consider that these patterns are less interesting for the new clinical knowledge, and that those patterns in which there is a significant change are those which are interesting in order to obtain surprising patterns.

With our new proposal, Jumping DOR Sequential Patterns (JDORSP), we therefore select only certain specific patterns using the non-overlapping of the DOR, that is, patterns that initially have a protection factor and whose extensions have a risk factor (41 patterns if we continue with the previous example) or the patterns that initially have a risk factor and whose extensions have a protection factor (49 patterns).

The same Table 7, therefore, shows that with JDORSP, expert discretization and 8% support, of the 163 patterns selected, there are 73 initial patterns and 90 extensions (41 with a risk factor and 49 with a protection factor).

### Technical discussion

Once a small number of sequential patterns that represent an abrupt change in patient evolution have been obtained, the interesting aspect is that of an expert manually evaluating each of the patterns obtained and attempting to explain their behavior. Table 8 shows some of the most interesting of the 38 patterns discovered (and their 45 extensions) using a 10% support with expert discretization. The full table is shown in Appendix A, in which the last two columns provide an evaluation by two clinicians of the level of interestingness of every pattern (surprisingness and relevance), using a scale of importance from 1 to 5 (not at all important, low importance, neutral, moderately important, very important). Firstly, Table A1 shows the patterns discovered that are initially at risk and whose extensions have a protection factor, while Table A2 shows the patterns that initially have a protection factor and are then at risk.

**Table 8:** Example of patterns discovered using JDORSP (Jumping DOR Sequential Patterns) mining process (all) with 10% support and expert discretization (extracted from Appendix A)

Num.	Pattern and extensions	SUR	DOR	DOR Interval	Patients	% Death	Meaning
(a) From Risk to Protection							
3	BAL_4_ < BIC_2_ < BIC_2_	1.81	2.16	(1.35, 3.45)	156	26.92% (42)	RISK
3A	BAL_4_ < BIC_2_ < BIC_2_ < PH_4_	1.81	0.35	(0.13, 0.95)	50	8% (4)	PROTECTION
13	INC_3_ < BE_2_ < BE_2_	1.51	1.76	(1.10, 2.81)	178	24.16% (43)	RISK
13A	INC_3_ < BE_2_ < BE_2_ < PH_4_	1.51	0.25	(0.08, 0.76)	50	6% (3)	PROTECTION
18	BAL_4_ < BIC_2_ < BE_2_	1.44	1.82	(1.14, 2.90)	185	24.32% (45)	RISK
18A	BAL_4_ < BIC_2_ < BE_2_ < PH_4_	1.44	0.38	(0.15, 0.96)	57	8.77% (5)	PROTECTION
22	BAL_4_ < BE_2_	1.37	1.88	(1.12, 3.17)	296	21.96% (65)	RISK
22A 22B	BAL_4_ < BE_2_ < INC_2_ BAL_4_ < BE_2_ < PH_4_	1.37 1.37	0.51 0.51	(0.27, 0.98) (0.29, 0.90)	102 139	11.76% (12) 12.23% (17)	PROTECTION PROTECTION
(b) From Protection to Risk							
34	PH_3_ < PH_3_ < PH_3_	3.47	0.59	(0.37, 0.94)	279	15.41% (43)	PROTECTION
34A 34B	PH_3_ < PH_3_ < PH_3_ < BAL_4_ PH_3_ < PH_3_ < PH_3_ = BE_1_	3.47 1.86	4.06 2.45	(1.85, 8.92) (1.03, 5.83)	24 23	45.83% (11) 34.78% (8)	RISK RISK
35	BIC_3_ < PH_3_	2.92	0.58	(0.36, 0.93)	249	14.86% (37)	PROTECTION
35A	BIC_3_ < PH_3_ < BAL_4_	2.92	3.50	(1.61, 7.60)	26	42.31% (11)	RISK
38	DIUR_2_ < PH_3_ < BIC_3_	1.77	0.61	(0.38, 0.98)	211	14.69% (31)	PROTECTION
38A	DIUR_2_ < PH_3_ < BIC_3_ < BAL_3_	1.77	2.38	(1.19, 4.76)	39	33.33% (13)	RISK

We have also calculated the absolute difference between the DOR value for the initial pattern and each extension. We believe that this score, which we have denominated as “SUR”, could be an indicator of the importance of the pattern extension in terms of surprisingness and relevance.

For example, one of the interesting patterns in Table 8 (extracted from Table A2) is pattern number 34 (PH_3_ < PH_3_ < PH_3_). This pattern has a statistically significant protection factor, with a DOR of 0.59 in the interval (0.37, 0.94). This pattern occurs for 279 patients, 43 of whom die (15.41%).

This pattern has the extension number 34A: PH_3_ < PH_3_ < PH_3_ < BAL_4_ with a DOR value of 4.06 in the interval (1.85, 8.92), signifying that it has a statistically significant risk factor. This pattern is found for 24 patients, 11 of whom die (45.83%). The surprisingness score (SUR) is 3.47 and is calculated as the absolute value of 0.59 minus 4.06.

It will, therefore, be noted that if the level of the PH is normal on three consecutive days, the patients will usually survive, but if the fluid balance is very high on the fourth day, then the patients have a much higher risk of death.

The use of a lower support makes it possible to discover patterns in which these changes are drastic. Table 9 shows the top 10 patterns discovered from Risk to Protection and the top 10 patterns discovered from Protection to Risk. These are ordered by SUR, using a 6% support with expert discretization (best) extracted from the 273 original JDORSP patterns discovered.

**Table 9:** Top 10 patterns discovered using JDORSP (Jumping DOR Sequential Patterns) mining process (best) with 6% support and expert discretization (some of them, extreme)

Pattern and extensions	SUR	DOR	DOR Interval	Patients	% Death	Meaning
(a) From Risk to Protection						
BIC_1_ < BE_2_ < BE_2_	2.56	2.65	(1.61, 4.36)	100	32% (32)	RISK
BIC_1_ < BE_2_ < BE_2_ < PH_4_	2.56	0.09	(0.01, 0.90)	22	0% (0)	PROTECTION
DIUR_3_ = BAL_4_ < DIUR_3_ = BIC_2_	2.50	2.58	(1.42, 4.67)	56	33.93% (19)	RISK
DIUR_3_ = BAL_4_ < DIUR_3_ = BIC_2_ < PH_4_	2.50	0.08	(0.01, 0.74)	25	0% (0)	PROTECTION
INC_3_ = DIUR_3_ < DIUR_3_ = BIC_2_	2.41	2.50	(1.38, 4.53)	57	33.33% (19)	RISK
INC_3_ = DIUR_3_ < DIUR_3_ = BIC_2_ < PH_4_	2.41	0.09	(0.01, 0.90)	22	0% (0)	PROTECTION
INC_3_ = DIUR_3_ = BAL_4_ < BIC_2_	2.32	2.48	(1.40, 4.39)	64	32.81% (21)	RISK
INC_3_ = DIUR_3_ = BAL_4_ < BIC_2_ < PH_4_	2.32	0.16	(0.03, 0.97)	26	3.85% (1)	PROTECTION
DIUR_3_ = BAL_4_ < BAL_4_	2.18	2.34	(1.39, 3.94)	88	30.68% (27)	RISK
DIUR_3_ = BAL_4_ < BAL_4_ < PH_4_	2.18	0.16	(0.03, 0.97)	26	3.85% (1)	PROTECTION
INC_3_ = DIUR_3_ < BIC_2_ = PH_3_	2.14	2.30	(1.30, 4.06)	67	31.34% (21)	RISK
INC_3_ = DIUR_3_ < BIC_2_ = PH_3_ < PH_4_	2.14	0.16	(0.03, 0.97)	26	3.85% (1)	PROTECTION
BIC_1_ < BE_2_	2.03	2.26	(1.42, 3.61)	157	27.39% (43)	RISK
BIC_1_ < BE_2_ < INC_2_	2.03	0.23	(0.06, 0.87)	37	5.41% (2)	PROTECTION
INC_3_ = DIUR_3_ < BIC_2_	1.98	2.09	(1.24, 3.51)	94	28.72% (27)	RISK
INC_3_ = DIUR_3_ < BIC_2_ < PH_4_	1.98	0.11	(0.02, 0.58)	37	2.7% (1)	PROTECTION
BE_0_ < DIUR_2_ < BAL_0_	1.97	2.06	(1.20, 3.53)	83	28.92% (24)	RISK
BE_0_ < DIUR_2_ < BAL_0_ = BE_3_	1.97	0.09	(0.01, 0.90)	22	0% (0)	PROTECTION
BAL_4_ < BE_2_ < BIC_2_ = BE_2_	1.93	2.13	(1.32, 3.42)	139	27.34% (38)	RISK
BAL_4_ < BE_2_ < BIC_2_ = BE_2_ < PH_4_	1.93	0.20	(0.05, 0.73)	40	4.76% (2)	PROTECTION
(b) From Protection to Risk						
BIC_2_ < DIUR_2_ = BAL_0_	45.55	0.49	(0.27, 0.88)	128	11.72% (15)	PROTECTION
BIC_2_ < DIUR_2_ = BAL_0_ < PH_1_	45.55	46.04	(8.24, 257.18)	5	100% (5)	RISK
DIUR_2_ < DIUR_2_ = BAL_0_	45.47	0.57	(0.34, 0.97)	156	13.46% (21)	PROTECTION
DIUR_2_ < DIUR_2_ = BAL_0_ < PH_1_	45.47	46.04	(8.24, 257.18)	5	100% (5)	RISK
PH_4_ < PH_4_	27.46	0.47	(0.26, 0.87)	123	11.38% (14)	PROTECTION
PH_4_ < PH_4_ < BE_1_	27.46	27.93	(6.71, 116.26)	7	85.71% (6)	RISK
DIUR_2_ = BAL_0_	27.41	0.52	(0.32, 0.87)	183	13.11% (24)	PROTECTION
DIUR_2_ = BAL_0_ < PH_1_	27.41	27.93	(6.71, 116.26)	7	85.71% (6)	RISK
DIUR_2_ < DIUR_2_ < BAL_0_	13.36	0.57	(0.34, 0.94)	176	13.64% (24)	PROTECTION
DIUR_2_ < DIUR_2_ < BAL_0_ = PH_1_	13.36	13.93	(3.97, 48.84)	8	75% (6)	RISK
INC_3_ = DIUR_2_ < BAL_0_	12.13	0.53	(0.30, 0.92)	143	12.59% (18)	PROTECTION
INC_3_ = DIUR_2_ < BAL_0_ = BE_0_	12.13	12.66	(4.34, 36.95)	11	72.73% (8)	RISK
DIUR_2_ < INC_2_ < BAL_0_	11.03	0.43	(0.22, 0.82)	107	10.28% (11)	PROTECTION
DIUR_2_ < INC_2_ < BAL_0_ = BE_0_	11.03	11.43	(3.04, 43.26)	7	71.43% (5)	RISK
BAL_0_ < BIC_3_	10.99	0.47	(0.27, 0.82)	153	11.76% (18)	PROTECTION
BAL_0_ < BIC_3_ = PH_2_	10.99	11.46	(3.04, 43.26)	7	71.43% (5)	RISK
DIUR_2_ < BE_2_ < PH_3_ < BIC_3_	10.99	0.47	(0.24, 0.91)	100	11% (11)	PROTECTION
DIUR_2_ < BE_2_ < PH_3_ < BIC_3_ < BAL_3_	10.99	11.46	(3.04, 43.26)	7	71.43% (5)	RISK
BIC_3_ < DIUR_2_	10.97	0.49	(0.31, 0.79)	237	13.5% (32)	PROTECTION
BIC_3_ < DIUR_2_ = BE_1_	10.97	11.46	(3.04, 43.26)	7	71.43% (5)	RISK

Note that there is a drastic change in the frequency properties of some of these patterns. This has made it possible to discover sequential patterns in which all the patients may eventually live or die. We call them as Extreme JDORSP.

For example, the pattern in which bicarbonate is low and base excess is normal later on 2 consecutive days (BIC_1_ < BE_2_ < BE_2_) has a statistically significant risk factor (DOR = 2.65). But, if the PH is a little higher the following day (BIC_1_ < BE_2_ < BE_2_ < PH_4_) then we have a statistically significant protection factor (DOR = 0.09), in which absolutely all the patients live (22 of the 22 patients that have this pattern live).

It is also necessary to observe patterns in which the change in frequency is very high (and not only 100%). If we observe the pattern PH_4_ < PH_4_, in which the PH is slightly higher for 2 days, it has a statistically significant protection factor, with DOR = 0.47, where only 14 out of 123 patients die (11.38%). But, if the base excess is low the following day, PH_4_ < PH_4_ < BE_1_, then we have a statistically significant risk factor, in which 6 out of 7 patients will die (85.71%).

### Discussion in the clinical study

In order to evaluate the level of interestingness (surprisingness and relevance) of the new sequential patterns discovered (JDORSP), the two clinicians chose to study the relationship between resuscitation related variables (fluid input and fluid balance), tissue perfusion related variables (arterial blood pH, bicarbonate concentration and base excess) and ICBU mortality. This decision was made for a number of reasons. Firstly, these are modifiable variables and, if an association with the outcome of interest is proven, a causal relationship could be hypothesized and, if this is proven, those variables could be used as therapeutic targets. Secondly, because those variables are related to the resuscitative efforts aimed at restoring organ perfusion after trauma. The fluids infused (in order to restore organ perfusion and urine output), the urine output (the most immediate goal of resuscitation), and the fluid balance (the difference between the fluids administered and the fluids lost by urine and other bodily losses), summarize the changes associated with the main therapeutic intervention immediately after trauma, i.e., fluid resuscitation. It could be said that the patterns regarding the patient’s evolution can have at least two different uses. In the first place, they can be used to establish therapeutic targets or outcomes to be achieved in the treatment of the patients, and in the second, they can be used as a means of monitoring and anticipating the appearance of risks in the patient.

The clinicians evaluated whether the sequential patterns add new knowledge (surprisingness) and whether they are clinically relevant because they may imply something interesting to review (relevance). Patterns will be good if they are relevant. If they are surprising, a possible line of interest for research could be identified, while if they are not novel but are confirmatory, it might be possible to conclude that the method could be used for other unexplored fields. A scale from 1 to 5 was used, where 1 is very low and 5 is very high. The relevance increases when it converts a risk factor into a protection factor, such as when there is a correction to an alteration, or when, after several abnormal determinations, a single corrected determination changes the prognosis.

Upon analyzing the tables in Appendix A, it will be noted that the patterns found are highly relevant, with an average relevance of 4.8. The novelty of the extensions is greater than that of the parent patterns, both globally and as regards the two types of patterns. With regard to the novelty that they provide, the extensions of the patterns that are transformed from a risk factor into a protection factor is very high (4.9) with respect to the global factor, which is 3.55, or the extensions of the patterns that become risky (3.36). In this case, it will be observed that the most interesting aspect is that after several days of being at risk, there is a change and the patterns start to have a protection factor. This change would not be surprising in other patterns with shorter duration.

The sequential patterns (of risk or protection) identified herein are of great clinical interest, as some are either very (scores close to 5) surprising or relevant. For instance, pattern 1, which indicates that a very positive fluid balance is associated with poor prognosis, is relevant, as it indicates that clinicians should take this change into account when prognosticating (and perhaps in order to fine tune fluid administration during resuscitation) burn patients. However, it is not surprising, as the currently accepted paradigm proposes that excessive fluid administration could lead to excessive edema formation and thus be associated with a poor prognosis. However, pattern 1A, which indicates that a strongly positive fluid balance followed by base excess and bicarbonate within the normal range, followed in turn by a pH rather in the alkalotic range, is protective is quite surprising. This is because it documents that the deleterious effects of a positive fluid balance appears to be offset if the pH is subsequently normalized (or even shifts towards the alkalotic range). This pattern is also relevant, as it reports the still incompletely known pathophysiology of the changes after trauma and their impact on prognosis.

## Conclusions and future work

This paper shows a proposal for a new method by which to obtain a reduced subset of surprising and innovative temporal patterns with which to represent the temporal evolution of a patient’s clinical state, denominated as Jumping Diagnostic Odds Ratio Sequential Patterns (JDORSP). The Diagnostic Odds Ratio (DOR) is used to select sequential patterns that represent a change in the evolution, that is, patterns that become a protection factor when we extend a pattern that was a risk factor, or vice versa. To the best of our knowledge, this is the first time that the DOR and sequential patterns have been used in this way.

We have evaluated the suitability of our method with patients in an Intensive Care Burns Unit. We highlight the drastic reduction in sequential patterns with respect to the current state of the art (Jumping Emerging Patterns or the non overlapping DOR). This remarkable reduction is particularly helpful for the subsequent manual review carried out by medical experts.

We have evaluated the surprisingness and relevance of the patterns with clinicians, and the most interesting fact is the high surprisingness (4.9 out of 5) of the sequential patterns that initially have a risk factor, and their extensions become a protection factor, that is, the patients that recover after several days of being at high risk of dying.

For further research we plan to explore other uses of the DOR and other epidemiological metrics, such as relative risk, as a measure of interestingness with which to calculate jumping sequential patterns. We are also working work on employing syntactic or semantic-based approaches to remove redundancy by coverage. A further post-process with which to improve the expressiveness of the patterns is also a research line and could, for example, be used to express very closely related patterns such as 14A and 14B (see Table A1).

### Electronic supplementary material

Below is the link to the electronic supplementary material.

Supplementary Material 1

## Data Availability

The datasets used and/or analysed during the current study are available from J.L. (joseangel.lorente@salud.madrid.org) on reasonable request.

## Abbreviations

BAL: balance of fluids
BE: base excess
BIC: bicarbonate
CI: confidence interval
DIUR: diuresis
DOR: Diagnostic Odds Ratio
EP: Emerging Pattern
FN: False negative
FP: False positive
ICBU: Intensive Care Burns Unit
INC: incomings
JDORSP: Jumping Diagnostic Odds Ratio Sequential Pattern
JEP: Jumping Emerging Pattern
TP: True positive
TN: True negative
