# Supplementary material for: Surprising and novel multivariate sequential patterns using odds ratio for temporal evolution in healthcare
Source: BMC Med Inform Decis Mak. 2024 Jun 13;24:165. doi: 10.1186/s12911-024-02566-4 (PMC11170878; doi:10.1186/s12911-024-02566-4)
Supplement: Supplementary file 1 — Supplementary Material 1 [file 12911_2024_2566_MOESM1_ESM.pdf]

Appendix A JDORSP discovered (with 10% support and expert discretization)

Table A1: The 59 patterns discovered, from RISK TO PROTECTION, using the Jumping DOR sequential pattern mining process with 10% support and expert discretization (all).

Num.	Pattern and extensions	SUR	DOR	DOR Interval	Patients	% Death	Meaning	Surpr.	Relev.
1	$BAL_4 < BE_2 < BIC_2 = BE_2$	1.93	2.13	(1.32, 3.42)	139	27.34% (38)	RISK	2	4
1A	$BAL_4 < BE_2 < BIC_2 = BE_2 < PH_4$	1.93	0.20	(0.05, 0.73)	42	4.76% (2)	PROTECTION	5	5
2	$INC_3 < BIC_2 = BE_2 < BE_2$	1.88	2.09	(1.29, 3.36)	136	27.21% (37)	RISK	4	4
2A	$INC_3 < BIC_2 = BE_2 < BE_2 < PH_4$	1.88	0.21	(0.06, 0.78)	40	5% (2)	PROTECTION	5	5
3	$BAL_4 < BIC_2 < BIC_2$	1.81	2.16	(1.35, 3.45)	156	26.92% (42)	RISK	2	4
3A	$BAL_4 < BIC_2 < BIC_2 < PH_4$	1.81	0.35	(0.13, 0.95)	50	8% (4)	PROTECTION	5	5
4	$BAL_4 < BE_2 < BIC_2$	1.77	1.94	(1.21, 3.12)	146	26.03% (38)	RISK	2	4
4A	$BAL_4 < BE_2 < BIC_2 < PH_4$	1.77	0.17	(0.05, 0.63)	47	4.26% (2)	PROTECTION	5	5
5	$DIUR_3 = BAL_4 < BIC_2$	1.74	1.84	(1.09, 3.11)	97	26.8% (26)	RISK	2	4
5A	$DIUR_3 = BAL_4 < BIC_2 < PH_4$	1.74	0.10	(0.02, 0.48)	42	2.38% (1)	PROTECTION	5	5
6	$BAL_4 < BIC_2$	1.72	2.18	(1.29, 3.69)	289	22.84% (66)	RISK	3	4
6A	$BAL_4 < BIC_2 < PH_4$	1.72	0.46	(0.26, 0.84)	132	11.36% (15)	PROTECTION	5	5
7	$DIUR_3 < BIC_2 = PH_3$	1.71	1.80	(1.10, 2.95)	120	25.83% (31)	RISK	5	4
7A	$DIUR_3 < BIC_2 = PH_3 < PH_4$	1.71	0.09	(0.02, 0.44)	45	2.22% (1)	PROTECTION	5	5
8	$DIUR_3 = BAL_4 < DIUR_3$	1.68	1.78	(1.06, 3.00)	99	26.26% (26)	RISK	2	4
8A	$DIUR_3 = BAL_4 < DIUR_3 < PH_4$	1.68	0.10	(0.02, 0.54)	39	2.56% (1)	PROTECTION	5	5
9	$BAL_4 < BIC_2 = BE_2 < BE_2$	1.65	1.91	(1.19, 3.06)	152	25.66% (39)	RISK	2	5
9A	$BAL_4 < BIC_2 = BE_2 < BE_2 < PH_4$	1.65	0.26	(0.08, 0.78)	49	6.12% (3)	PROTECTION	5	5
10	$BAL_4 < BIC_2 = BE_2 < BIC_2$	1.63	1.73	(1.05, 2.86)	118	25.42% (30)	RISK	2	5
10A	$BAL_4 < BIC_2 = BE_2 < BIC_2 < PH_4$	1.63	0.10	(0.02, 0.50)	41	2.44% (1)	PROTECTION	5	5
11	$BAL_4 < BIC_2 < BIC_2 = BE_2$	1.62	1.92	(1.19, 3.11)	133	26.32% (35)	RISK	3	5
11A	$BAL_4 < BIC_2 < BIC_2 = BE_2 < PH_4$	1.62	0.30	(0.10, 0.94)	43	6.98% (3)	PROTECTION	5	5
12	$BAL_4 < BE_2 < BE_2$	1.61	1.90	(1.19, 3.03)	202	24.26% (49)	RISK	2	5
12A	$BAL_4 < BE_2 < BE_2 < PH_4$	1.61	0.29	(0.11, 0.78)	58	6.9% (4)	PROTECTION	5	5
13	$INC_3 < BE_2 < BE_2$	1.51	1.76	(1.10, 2.81)	178	24.16% (43)	RISK	3	5
13A	$INC_3 < BE_2 < BE_2 < PH_4$	1.51	0.25	(0.08, 0.76)	50	6% (3)	PROTECTION	5	5
14	$BAL_4 < BAL_4 < DIUR_2$	1.50	1.68	(1.05, 2.69)	172	23.84% (41)	RISK	2	5
14A	$BAL_4 < BAL_4 < DIUR_2 < PH_4$	1.50	0.18	(0.05, 0.67)	45	4.44% (2)	PROTECTION	5	5
14B	$BAL_4 < BAL_4 < DIUR_2 = PH_4$	1.45	0.23	(0.08, 0.69)	54	5.56% (3)	PROTECTION	5	5
15	$BIC_2 < BIC_2$	1.46	1.92	(1.17, 3.14)	263	22.81% (60)	RISK	3	5

15A	$BIC_2 < BIC_2 < PH_4$	1.46	0.46	(0.24, 0.87)	110	10.91% (12)	PROTECTION	5	5
16	$INC_3 < BIC_2 = BE_2$	1.46	1.88	(1.18, 2.99)	203	24.14% (49)	RISK	3	5
16A	$INC_3 < BIC_2 = BE_2 < INC_2$	1.46	0.42	(0.20, 0.88)	82	9.76% (8)	PROTECTION	5	5
17	$BAL_4 < DIUR_2$	1.45	1.90	(1.05, 3.43)	343	20.99% (72)	RISK	2	5
17A	$BAL_4 < DIUR_2 = PH_4$	1.45	0.45	(0.25, 0.82)	134	11.19% (15)	PROTECTION	5	5
17B	$BAL_4 < DIUR_2 < PH_4$	1.44	0.46	(0.26, 0.81)	147	11.56% (17)	PROTECTION	2	5
18	$BAL_4 < BIC_2 < BE_2$	1.44	1.82	(1.14, 2.90)	185	24.32% (45)	RISK	2	5
18A	$BAL_4 < BIC_2 < BE_2 < PH_4$	1.44	0.38	(0.15, 0.96)	57	8.77% (5)	PROTECTION	5	5
19	$BAL_4 < BAL_4 = PH_3$	1.44	1.76	(1.09, 2.83)	149	24.83% (37)	RISK	2	5
19A	$BAL_4 < BAL_4 = PH_3 < PH_4$	1.44	0.32	(0.12, 0.86)	54	7.41% (4)	PROTECTION	5	5
20	$BAL_4 = BIC_2 < BIC_2$	1.42	1.70	(1.06, 2.73)	161	24.22% (39)	RISK	2	5
20A	$BAL_4 = BIC_2 < BIC_2 < PH_4$	1.42	0.28	(0.12, 0.69)	72	6.94% (5)	PROTECTION	5	5
21	$INC_3 < BAL_4 < DIUR_2$	1.42	1.63	(1.01, 2.64)	150	24% (36)	RISK	2	5
21A	$INC_3 < BAL_4 < DIUR_2 < PH_4$	1.42	0.21	(0.06, 0.78)	40	5% (2)	PROTECTION	5	5
22	$BAL_4 < BE_2$	1.37	1.88	(1.12, 3.17)	296	21.96% (65)	RISK	2	5
22A	$BAL_4 < BE_2 < INC_2$	1.37	0.51	(0.27, 0.98)	102	11.76% (12)	PROTECTION	5	5
22B	$BAL_4 < BE_2 < PH_4$	1.37	0.51	(0.29, 0.90)	139	12.23% (17)	PROTECTION	5	5
23	$BAL_4 < BAL_3$	1.35	1.71	(1.06, 2.73)	228	22.81% (52)	RISK	2	5
23A	$BAL_4 < BAL_3 < PH_4$	1.35	0.36	(0.17, 0.76)	91	8.79% (8)	PROTECTION	5	5
24	$INC_1 < BIC_2$	1.35	1.64	(1.004, 2.68)	131	24.43% (32)	RISK	4	5
24A	$INC_1 < BIC_2 < BIC_3$	1.35	0.29	(0.09, 0.88)	45	6.67% (3)	PROTECTION	5	5
25	$INC_3 < BAL_4 < PH_3$	1.33	1.61	(1.002, 2.57)	166	23.49% (39)	RISK	2	5
25A	$INC_3 < BAL_4 < PH_3 < PH_4$	1.33	0.28	(0.09, 0.86)	46	6.52% (3)	PROTECTION	5	5
26	$DIUR_3 < BIC_2$	1.31	1.60	(1.003, 2.57)	171	23.39% (40)	RISK	4	5
26A	$DIUR_3 < BIC_2 < PH_4$	1.31	0.29	(0.12, 0.70)	71	7.04% (5)	PROTECTION	5	5
27	$BAL_4 < BIC_2 = PH_3$	1.29	1.63	(1.02, 2.60)	216	22.69% (49)	RISK	2	5
27A	$BAL_4 < BIC_2 = PH_3 < PH_4$	1.29	0.34	(0.16, 0.71)	95	8.42% (8)	PROTECTION	5	5
28	$BAL_4 < BIC_2 = PH_3 = BE_2$	1.17	1.60	(1.003, 2.57)	171	23.39% (40)	RISK	2	5
28A	$BAL_4 < BIC_2 = PH_3 = BE_2 < PH_4$	1.17	0.43	(0.20, 0.91)	80	10% (8)	PROTECTION	5	5

Table A2: The 24 patterns discovered, from PROTECTION to RISK, using the Jumping DOR sequential pattern mining process with 10% support and expert discretization (all).

Num.	Pattern and extensions	SUR	DOR	DOR Interval	Patients	% Death	Meaning	Surpr.	Relev.
29	$INC_3 = DIUR_2 < BAL_0$	12.13	0.53	(0.30, 0.92)	143	12.59% (18)	PROTECTION	2	5
29A	$INC_3 = DIUR_2 < BAL_0 = BE_0$	12.13	12.66	(4.34, 36.95)	11	72.73% (8)	RISK	3	5
30	$DIUR_2 < DIUR_2 < BAL_0$	12.09	0.57	(0.34, 0.94)	176	13.64% (24)	PROTECTION	4	5
30A	$DIUR_2 < DIUR_2 < BAL_0 = BE_0$	12.09	12.66	(4.34, 36.95)	11	72.73% (8)	RISK	3	5
31	$DIUR_2 < BAL_0$	10.22	0.58	(0.36, 0.93)	275	15.27% (42)	PROTECTION	2	5
31A	$DIUR_2 < BAL_0 = BE_0$	10.22	10.80	(4.42, 26.39)	16	68.75% (11)	RISK	3	5
31B	$DIUR_2 < BAL_0 = BIC_1$	5.43	6.01	(2.54, 14.20)	18	55.56% (10)	RISK	3	5
31C	$DIUR_2 < BAL_0 = BE_1$	2.51	3.09	(1.27,7.50)	20	40.00% (8)	RISK	3	5
32	$DIUR_2 = PH_3$	5.69	0.59	(0.36, 0.96)	346	16.47% (57)	PROTECTION	3	5
32A	$DIUR_2 = PH_3 < PH_1$	5.69	6.28	(2.40, 16.44)	14	57.14% (8)	RISK	3	5
32B	$DIUR_2 = PH_3 < BE_0$	2.04	2.63	(1.10, 6.30)	22	36.36% (8)	RISK	3	5
33	$BIC_3 = PH_3 < PH_3$	3.67	0.58	(0.35, 0.95)	192	14.06% (27)	PROTECTION	4	5
33A	$BIC_3 = PH_3 < PH_3 < BAL_4$	3.67	4.25	(1.78, 10.11)	19	47.37% (9)	RISK	5	5
34	$PH_3 < PH_3 < PH_3$	3.47	0.59	(0.37, 0.94)	279	15.41% (43)	PROTECTION	2	5
34A	$PH_3 < PH_3 < PH_3 < BAL_4$	3.47	4.06	(1.85, 8.92)	24	45.83% (11)	RISK	3	5
34B	$PH_3 < PH_3 < PH_3 = BE_1$	1.86	2.45	(1.03, 5.83)	23	34.78% (8)	RISK	3	5
35	$BIC_3 < PH_3$	2.92	0.58	(0.36, 0.93)	249	14.86% (37)	PROTECTION	2	5
35A	$BIC_3 < PH_3 < BAL_4$	2.92	3.50	(1.61, 7.60)	26	42.31% (11)	RISK	5	5
36	$BIC_3 = PH_3$	2.19	0.39	(0.24, 0.62)	285	12.98% (37)	PROTECTION	2	5
36A	$BIC_3 = PH_3 < PH_2$	2.19	3.09	(1.27, 7.50)	20	40% (8)	RISK	3	5
37	$PH_3 < BIC_3$	1.99	0.46	(0.29, 0.74)	309	14.56% (45)	PROTECTION	2	5
37A	$PH_3 < BIC_3 = PH_2$	1.99	2.45	(1.03, 5.83)	23	34.78% (8)	RISK	3	5
38	$DIUR_2 < PH_3 < BIC_3$	1.77	0.61	(0.38, 0.98)	211	14.69% (31)	PROTECTION	3	5
38A	$DIUR_2 < PH_3 < BIC_3 < BAL_3$	1.77	2.38	(1.19, 4.76)	39	33.33% (13)	RISK	4	5
